# Recent Metabolic Advances for Preventing and Treating Acute and Chronic Graft Versus Host Disease

**DOI:** 10.3389/fimmu.2021.757836

**Published:** 2021-10-12

**Authors:** Fathima A. Mohamed, Govindarajan Thangavelu, Stephanie Y. Rhee, Peter T. Sage, Roddy S. O’Connor, Jeffrey C. Rathmell, Bruce R. Blazar

**Affiliations:** ^1^ Department of Pediatrics, Division of Blood & Marrow Transplant & Cellular Therapy, University of Minnesota Cancer Center, Minneapolis, MN, United States; ^2^ Renal Division, Transplantation Research Center, Brigham and Women’s Hospital, Harvard Medical School, Boston, MA, United States; ^3^ Department of Pathology and Laboratory Medicine, University of Pennsylvania, Philadelphia, PA, United States; ^4^ Center for Cellular Immunotherapies, Perelman School of Medicine, Philadelphia, PA, United States; ^5^ Department of Pathology, Microbiology, and Immunology, Vanderbilt University Medical Center, Nashville, TN, United States

**Keywords:** metabolism, graft-versus-host disease, intestinal epithelial cells, alloreactive T-cells, graft-versus-tumor

## Abstract

The therapeutic efficacy of allogeneic hematopoietic stem cell transplantation (allo-HSCT) is limited by the development of graft-versus-host disease (GVHD). In GVHD, rigorous pre-conditioning regimen resets the immune landscape and inflammatory milieu causing immune dysregulation, characterized by an expansion of alloreactive cells and a reduction in immune regulatory cells. In acute GVHD (aGVHD), the release of damage- and pathogen- associated molecular patterns from damaged tissue caused by the conditioning regimen sets the stage for T cell priming, activation and expansion further exacerbating tissue injury and organ damage, particularly in the gastrointestinal tract. Studies have shown that donor T cells utilize multiple energetic and biosynthetic pathways to mediate GVHD that can be distinct from the pathways used by regulatory T cells for their suppressive function. In chronic GVHD (cGVHD), donor T cells may differentiate into IL-21 producing T follicular helper cells or tissue resident T helper cells that cooperate with germinal center B cells or memory B cells, respectively, to produce allo- and auto-reactive antibodies with subsequent tissue fibrosis. Alternatively, donor T cells can become IFN- γ/IL-17 cytokine expressing T cells that mediate sclerodermatous skin injury. Patients refractory to the first line standard regimens for GVHD treatment have a poor prognosis indicating an urgent need for new therapies to restore the balance between effector and regulatory immune cells while preserving the beneficial graft-versus-tumor effect. Emerging data points toward a role for metabolism in regulating these allo- and auto-immune responses. Here, we will discuss the preclinical and clinical data available on the distinct metabolic demands of acute and chronic GVHD and recent efforts in identifying therapeutic targets using metabolomics. Another dimension of this review will examine the changing microbiome after allo-HSCT and the role of microbial metabolites such as short chain fatty acids and long chain fatty acids on regulating immune responses. Lastly, we will examine the metabolic implications of coinhibitory pathway blockade and cellular therapies in allo-HSCT. In conclusion, greater understanding of metabolic pathways involved in immune cell dysregulation during allo-HSCT may pave the way to provide novel therapies to prevent and treat GVHD.

## Introduction

Allogeneic hematopoietic stem cell transplantation (allo-HSCT) is an effective and widely used cellular therapy for various malignant and benign hematological disorders. The beneficial effect of allo-HSCT is dependent on donor T cells which promote bone marrow engraftment and mediate the graft-versus-tumor (GVT) effect against residual cancer cells that survive conditioning regimen. However, the downside of donor T cell alloreactivity can be graft-versus-host disease (GVHD) which involves the attack of histocompatibility-disparate healthy recipient tissues and is the most common cause of non-relapse morbidity and mortality after allo-HSCT (1, 2).

**Figure 1:**
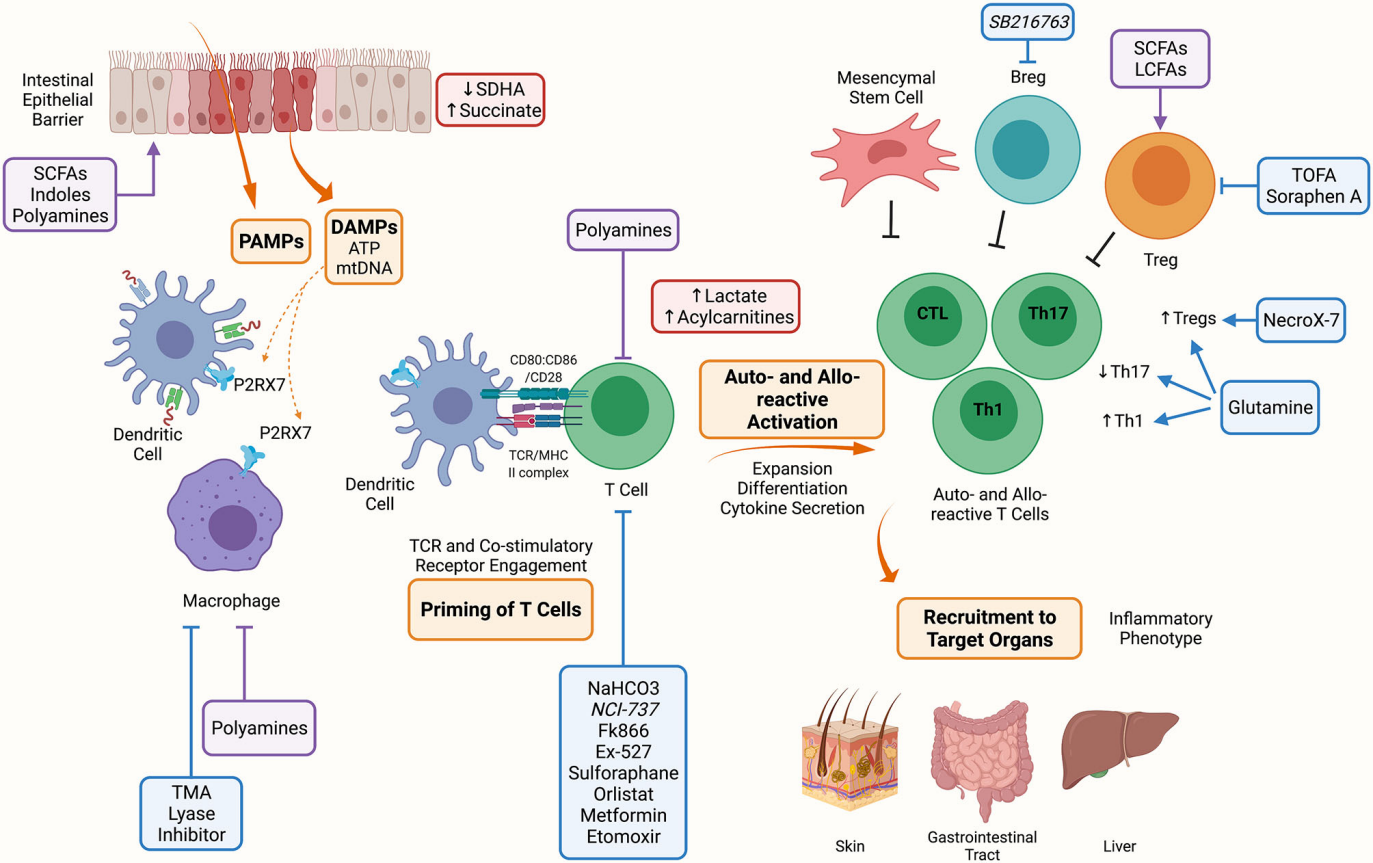
Metabolic Targets of Acute Graft Versus Host Disease. The conditioning regimen mediates tissue damage releasing PAMPs and DAMPs, these prime APCs to activate T cells. Alloreactive T cells expand and differentiate into inflammatory Th1, Th17 and CTLs. Alloreactive T cells contribute to the inflammatory milieu through secretion of IL-2, IFN-γ and TNF-a. These activated alloreactive donor T cells can be recruited to target organs including the skin, GI tract and liver to mediate the inflammatory phenotype characteristic of aGVHD. Indicated in blue are metabolic targets for the treatment of aGVHD. Trimethylamine (TMA) lyase inhibitor prevents the polarization of macrophages into M1 cells. Inhibitors against the nicotinamide phosphoribosyl transferase pathway (Fk866), lactate dehydrogenase (NCI-737), sirtuin-1 (Ex-527), lysosomal lipase (orlistat), fatty acid oxidation (etomoxir); along with activators of 5’-AMP-activated protein kinase (AMPK) (metformin) and nuclear factor 2 (sulforaphane) may or have been shown to target allo-reactive T cell function in aGVHD. NaHCO_3_ has been shown to abrogate lactate accumulation in allo-reactive T cells. Inhibitors of fatty acid synthesis (FAS) (5-(Tetradecyloxy)-2-furoic acid (TOFA), Soraphen A), reactive oxygen species (NecroX-7), and glutamine administration improve the suppressive function and increase Tregs aiding to alleviate aGVHD. Lastly, GSK3β inhibitor (SB216763) has been shown to enhance regulatory B cells (Bregs) differentiation and suppressive function and may be a therapeutic target in aGVHD. Indicated in red are metabolites that have been shown to accumulate during aGVHD, these include succinate in IECs due to reduced SDHA activity, and lactate and acylcarnitines in allo-reactive T cells. Indicated in purple are microbial metabolites that act on cells during aGVHD, these include short chain fatty acids (SCFAs), long chain fatty acids (LCFAs), indoles and polyamines which can act on immune (macrophages, T cells, Tregs) and non-immune (IECs) cells. Created with BioRender.com.

Acute GVHD (aGVHD) generation requires a multi-step process (**Figure 1**). Proinflammatory early events begin with a conditioning regimen-mediated tissue injury that causes the release of inflammatory triggers, such as damage associated molecular patterns (DAMPs) and pathogen-associated molecular patterns (PAMPs). Inflammation rapidly recruits host cells of the innate and adaptive immune system that contribute to tissue injury and activation of antigen-presenting cells (APCs) of donor and host origin that prime the adaptive immune response. Following activation, alloreactive donor T cells expand, and differentiate into effector cells that secrete proinflammatory cytokines (2, 3). Effector cells are recruited to target organs where cytokines (i.e., IFN- γ) can cause tissue damage such as intestinal stem cell injury in the gastrointestinal tract and cytotoxic molecules (i.e., perforin) amplify end organ damage (2, 3).

**Figure 2:**
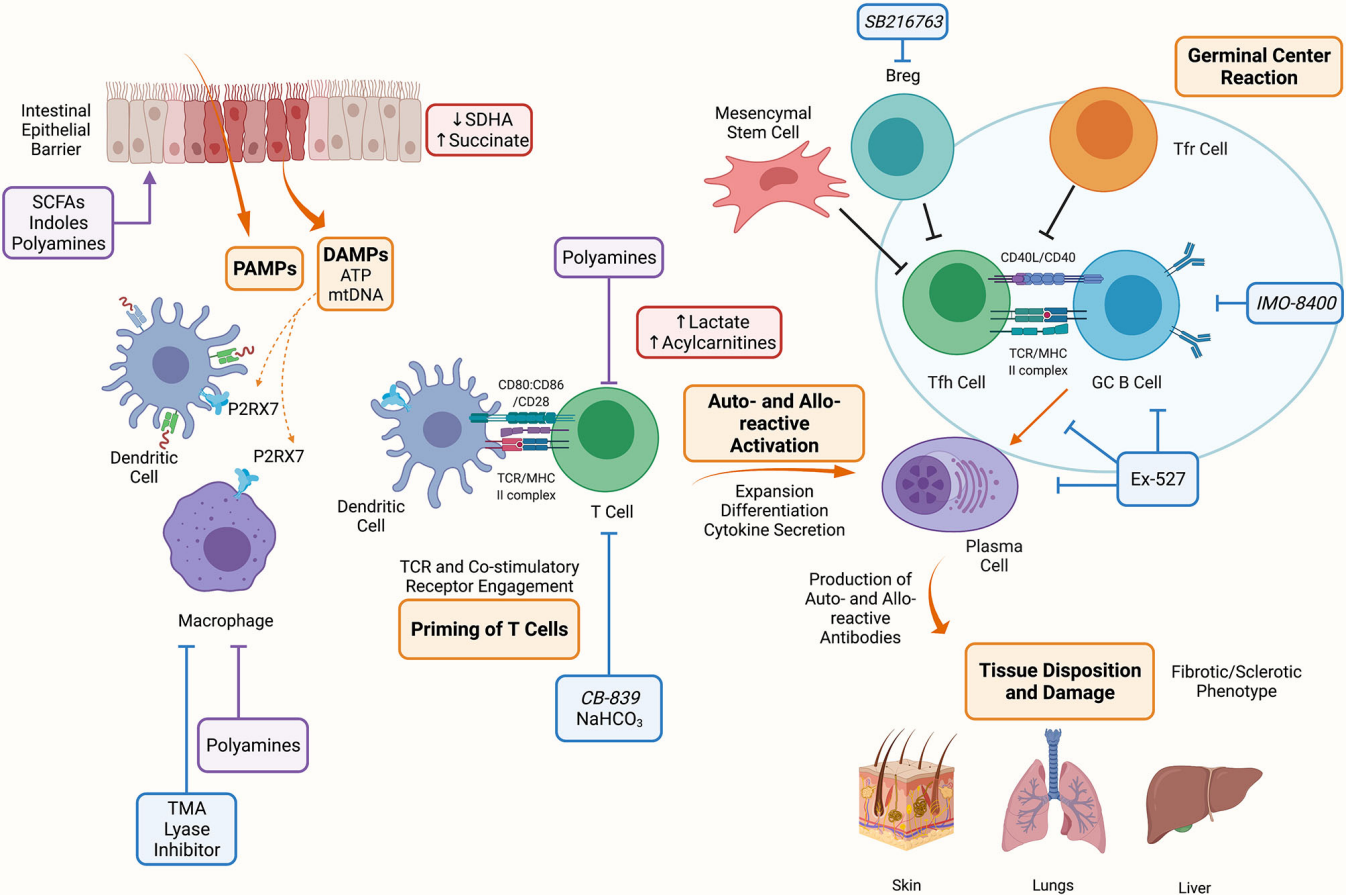
Metabolic Targets of Chronic Graft Versus Host Disease. The conditioning regimen mediates tissue damage releasing PAMPs and DAMPs, these prime APCs to activate T cells. Alloreactive T cells expand and differentiate into Tfh cells which provide costimulatory signals to B cells resulting in the formation of the germinal center and production of auto- and allo-antibodies. These antibodies deposit onto target organs such as skin, lungs and liver mediating the fibrotic and sclerotic phenotype characteristic of cGVHD. Indicated in blue are metabolic targets for the treatment of cGVHD. Trimethylamine (TMA) lyase inhibitor prevents the polarization of macrophages into M1 cells. NaHCO_3_ has been shown to abrogate lactate accumulation in allo-reactive T cells. CB-839 is a glutaminase inhibitor that may be effective in cGVHD since GLS deficient T cells are unable to establish disease. Ex-527 is an inhibitor against Sirtuin-1 that reduced Tfh cell, B cell and plasma cell differentiation and IMO-8400 is a TLR 7/8/9 inhibitor that may be effective against B cells in cGVHD. Lastly, SBGSK3β inhibitor (SB216763) has been shown to enhance regulatory B cells (Bregs) differentiation and suppressive function and may be a therapeutic target in cGVHD. Indicated in red are metabolites that have been shown to accumulate during aGVHD, these include succinate in IECs due to reduced SDHA activity, and lactate and acylcarnitines in allo-reactive T cells. Indicated in purple are microbial metabolites that act on or influence the function of cells during cGVHD, these include short chain fatty acids (SCFAs), long chain fatty acids (LCFAs), indoles and polyamines which can act on immune (macrophages, T cells) and non-immune (IECs) cells. Created with BioRender.com.

Similar to aGVHD, chronic GVHD (cGVHD) is initiated by conditioning regimen injury and subsequent inflammation. Alloreactive T- and B- cells are activated by host APCs and T cells. T cells are polarized to Th1, Th2, or Th17 cells. Auto- and allo- reactive T cells escape deletion due to thymic injury and deficient generation of thymus-derived regulatory T cells (Tregs) leads to low numbers of peripheral Tregs, T follicular regulatory (Tfr) cells and immune dysregulation (**Figure 2**). Under these conditions, activated T cells can differentiate into T follicular helper (Tfh) or pre-Tfh cells that secrete IL-21 or IL-17, and signal B cells to produce auto- and allo- antibodies that are deposited in cGVHD target organs and contribute to further tissue injury and chemokines release (4). Fc receptors on recruited monocytes and macrophages are ligated by deposited immunoglobulin, stimulating fibroblasts to secrete extracellular matrix components favoring fibrosis that cause an obstructive lung disease known as bronchiolitis obliterans (1).

Despite differences in pathophysiology between aGVHD, characterized by a cellular tissue destructive process, and cGVHD characterized by T cell: B cell cooperativity, antibody deposition and fibrosis, corticosteroids are the first line therapy for both GVHD types. Steroids are broadly immunosuppressive, have considerable side-effects and increase susceptibility to opportunistic infections. Steroid refractory patients have a poor prognosis (2) highlighting the need to continue to pursue novel therapies to control GVHD, retain GVT response and avoid broad immune suppression. Recently strategies targeting metabolic pathways in immune cell populations have been garnering attention given the specialized substrate and energy requirements of immune cell types. The high proliferative, differentiation and migratory needs of GVHD-causing T- and B- cells; and of cells involved in tissue repair depend upon sufficient substrate availability that can be used by metabolic pathways for robust ATP production, biosynthesis, and reduction-oxidation (redox) reactions. Since these processes occur in different environments by distinct cell types, there is a therapeutic opportunity to inhibit GVHD pathogenesis while favoring cell repair mechanisms.

## Metabolic Pathways Known to Contribute to GVHD

Studies have linked glycolysis, fatty acid synthesis (FAS), and glutaminolysis to GVHD. During glycolysis, which occurs in the cytoplasm, glucose is broken down to generate two molecules of pyruvate and ATP (5). Pyruvate can enter the tricarboxylic acid (TCA) cycle to fuel ATP production or to support TCA cycle anaplerosis. The first step of the TCA cycle is the formation of citrate from acetyl-CoA and oxaloacetate. A complete turn of the TCA cycle yields GTP, CO_2_ and reduced forms of nicotinamide adenine dinucleotide (NAD) + hydrogen (NADH) and flavin adenine dinucleotide (FADH_2_). These each can be used as a cofactor for use by multiple enzymes or to shuttle electrons into the mitochondrial electron transport chain (ETC) Complex I and Complex II. The final electron acceptor is O_2_; high amounts of ATP are produced in a process known as oxidative phosphorylation (OXPHOS) (5).

Highly proliferative cells can preferentially rely on ATP from cytosolic glycolysis instead of mitochondrial TCA cycle through a process called aerobic glycolysis. Also known as the Warburg effect (6), pyruvate is converted into lactate by lactate dehydrogenase (LDH), replenishing NAD+ that is required for glycolysis. Alternatively, pyruvate is decarboxylated by pyruvate dehydrogenase complex (PDH) to form acetyl-CoA that enters the TCA cycle eventually feeding into the mitochondrial ETC. Thus, PDH regulates the metabolic finetuning between glycolysis and FAO through regulation of acetyl-CoA.

Glycolysis also produces intermediates for the downstream synthesis of nucleotides and amino acids *via* the pentose phosphate pathway (PPP) and nicotinamide adenine dinucleotide phosphate + hydrogen (NADPH) for protection against oxidative stress (5). Metabolic reprogramming of T cells and other immune cell types to use of metabolic pathways that optimally exploit available substrates may prove advantageous by rapidly generating ATP in a substrate limited environment or promoting glycolytic intermediate flux into biosynthetic pathways (7). TCA cycle intermediates also can serve as connection points to multiple metabolic processes. For example, TCA intermediates, including citrate, itaconate, succinate, fumarate, and L-malate, can accumulate and regulate pro- and anti-inflammatory gene expression in immune cells (8). In the setting of cellular stress TCA intermediates can be released from the mitochondria and act as regulators of the immune system (8). Such intermediates promote reactive oxygen species (ROS) production that can signal inflammatory responses and mediate post-translational modification of metabolic pathway enzymes. The export of citrate and succinate from the mitochondria is linked to the production of pro-inflammatory mediators in macrophages including ROS, nitric oxide, and prostaglandin E2 (8, 9). Alpha ketoglutarate can regulate NF-kB signaling mediating pro-inflammatory responses (10) and mutations in the alpha ketoglutarate generating enzyme, isocitrate dehydrogenase (IDH), are associated with diseases of chronic inflammation (11). Conversely, itaconate, alpha ketoglutarate and fumarate enhance immunosuppression (8). Overall, TCA intermediates can have pro and/or anti-inflammatory effects.

**Figure 3:**
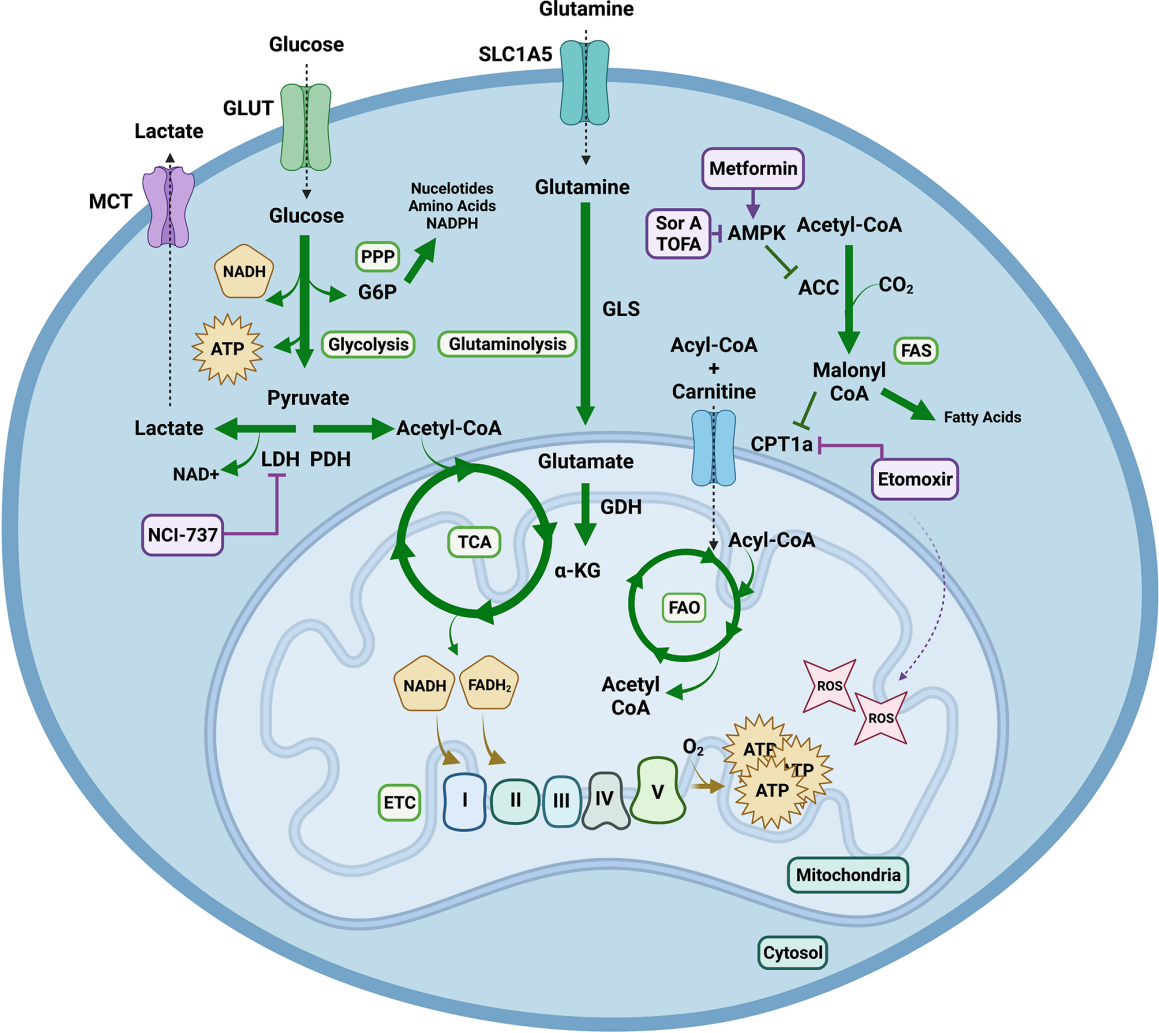
Metabolic Pathways Contributing to Graft Versus Host Disease. Extracellular glucose is transported into the cell and utilized to generate two molecules of pyruvate and ATP in glycolysis. Pyruvate has multiple fates. It can be converted into lactate *via* lactate dehydrogenase (LDH) replenishing nicotinamide adenine dinucleotide (NAD) + for glycolysis. This can be exploited by highly proliferative cells to continuously produce ATP *via* glycolysis, even in the presence of O_2_, in a process known as aerobic glycolysis or the Warburg effect. Lactate can be exported from the cell through monocarboxylic transporters (MCT). Alternatively, pyruvate can be decarboxylated into acetyl-CoA *via* pyruvate dehydrogenase (PDH) to enter the tricarboxylic acid (TCA) cycle. One turn of the TCA cycle yields GTP, CO_2_ and reduced forms of nicotinamide adenine dinucleotide hydrogen (NADH) and flavin adenine dinucleotide (FADH_2_); which can shuttle electrons into the mitochondrial electron transport chain (ETC) where O_2_ serves as the final electron acceptor to generate high amounts of ATP in a process known as oxidative phosphorylation (OXPHOS). OXPHOS can mediate production of reactive oxygen species (ROS) such as superoxide. During glutaminolysis glutamine is hydrolyzed into glutamate, which can be converted by glutamate dehydrogenase (GDH) into alpha ketoglutarate (α-KG) in order to enter the TCA cycle. Additionally, fatty acids (FA) undergo beta oxidation in the mitochondrial matrix to produce acetyl-CoA and electron carriers. The formation of malonyl-CoA from acetyl-CoA by acetyl-CoA carboxylase (ACC) early in fatty acid synthesis (FAS), inhibits carnitine palmitoyltransferase (CPT1a), the rate limiting step of fatty acid oxidation (FAO), strictly controlling the processes of FAS and FAO. Lastly, the nuclear energy sensor 5’-AMP-activated protein kinase (AMPK) can inhibit ACC releasing inhibition of CPT1a thus indirectly promoting FAO. Pharmacological agents targeting these metabolic pathways include NCI-737 inhibiting LDHA, metformin activating AMPK, soraphen A and 5-(Tetradecyloxy)-2-furoic acid (TOFA) inhibiting ACC and etomoxir inhibiting CPT1a. Notably concentrations of etomoxir above 5uM have been shown to induce ROS production. Created with BioRender.com.

Fatty acid oxidation (FAO) is linked to the FA beta-oxidation that occurs in the mitochondrial matrix, wherein lipids are metabolized to produce acetyl-CoA and electron carriers. The formation of malonyl-CoA from acetyl-CoA by acetyl-CoA carboxylase (ACC) early in FAS, inhibits carnitine palmitoyltransferase (CPT1a), the rate limiting step of FAO, strictly controlling the processes of FAS and FAO (12). The intricate balance of these metabolic pathways serves to support the effector function of rapidly proliferating, expanding and differentiating immune cells (**Figure 3**).

## Modulating Donor T Cell Metabolism to Control GVHD and Preserve GVT

### Glycolysis and Glutaminolysis

T cell function, including the ability of T cells to eliminate tumor cells, depends on their mitochondrial fitness (13). A recent study in allo-HSCT divulged the critical role of glycolytic activity of T cells in mediating GVT responses (14). Tumors evade immune surveillance by downregulating antigen presentation, secreting soluble cytokines, recruiting Tregs to the tumor milieu, and promoting factors that support immune tolerance and immune evasion (15). Leukemic cells can also evade T cell lysis by creating a distinct environment containing immune suppressive metabolites including lactic acid. T cells isolated from the same allo-HSCT patients exhibited distinct metabolic phenotypes based on the status of tumor relapse. T cells harvested during tumor relapse exhibited reduced glycolytic activity and OXPHOS compared to those harvested during remission. These findings correlated with increased serum lactic acid levels in tumor-relapsing patients. Mechanistically, production of lactic acid by tumor cells impaired T cell metabolic fitness, proliferation, and cytokine production and thus reduced GVT responses. At physiologic pH, lactic acid dissociates into H+ and La^-^. Using ^13^C lactate and LC/MS approaches, the authors showed that lactate is consumed by proliferating T cells in an MCT-1-dependent manner and integrated into their TCA cycle. Although speculative, lactate consumed in this manner may impede T cell function by end-point inhibition of glycolysis or an increase in reductive stress by skewing NADH/NAD+ ratios towards excess NADH accumulation. Interestingly their data suggests that sustained exposure to elevated lactate above a critical threshold of 10mM (which didn’t impede T cell function) whereas levels >15mM) impaired T cell function. Administration of sodium bicarbonate (NaHCO_3_) to counteract acidosis, restored GVT responses in mouse tumor models by rescuing T cell metabolic fitness and function. Interestingly, a short-term treatment of NaHCO_3_ in allo-HSCT patients increased their T cells’ respiratory capacity and effector cytokine production. These findings provide evidence that the metabolic reprogramming of donor T cells may be exploited to enhance their GVT activity in patients with tumor relapse.

By employing novel, noninvasive hyperpolarized ^13^C-pyruvate magnetic resonance imaging (MRI) Assmann et al. (16) were able to diagnose GVHD prior to the onset of clinical manifestations in a mouse model of cGVHD. Imaging analysis at an early time-point identified that allo-HSCT mice had a higher conversion of pyruvate into lactate in the liver than those of syngeneic controls. However, no difference was observed at later timepoints which may be due to the change in metabolic shifts and reduced T cell activity. Further analysis using transcriptomic, metabolite and *ex vivo* metabolic activity assays demonstrated that pathogenic donor CD4+ T cells were highly glycolytic. Single cell sequencing of circulating CD4+ T cells isolated from two allo-HSCT patients revealed similar metabolic changes due to the increased transcription of glycolytic enzymes even before the onset of aGVHD clinical signs (16). Overall, this novel technique is informative in assessing the onset of GVHD and pointing to reducing glycolysis and “starving” GVHD pathogenic cells. Direct targeting of glycolysis can be achieved by administering an inhibitor such as NCI-737 to lactate dehydrogenase A (LDHA), the main enzyme responsible for the Warburg effect that has shown promise as an anti-cancer agent in preclinical trials (17). Metformin is an FDA approved biguanide with a low toxicity profile and is in widespread use as a treatment for type 2 diabetes and metabolic syndrome. Metformin lowers glucose by increasing insulin sensitivity, decreasing absorption, and blocking gluconeogenesis. Metformin effects on 5’-AMP-activated protein kinase (AMPK) are discussed in 3.3 below.

Activated effector T cells also need to increase their glutamine uptake to allow for adequate ATP production by glutaminolysis (18). Glutamine is a conditionally essential amino acid in proliferating cells and is hydrolyzed by glutaminase (GLS) to produce glutamate. Glutamate can be converted by glutamate dehydrogenase to alpha ketoglutarate to enter the TCA cycle or when combined with cysteine and glycine to form glutathione, an antioxidant that protects cells from detrimental redox reactions. Our recent study demonstrated that GLS deficiency impaired Th17 and promoted Th1 cell differentiation that was associated with altered gene expression and chromatin accessibility. GLS deficient T cells were unable to drive Th17 mediated inflammatory diseases (18). Relevant to GVHD, other reports have implicated Th17 cells in murine aGVHD pathogenesis (18), and a Th17-prone population in pre-symptomatic aGVHD patients (19). In aGVHD patients, a CD146+CCR5+ Th17-prone cell population correlated with disease (19). Glutamine administration in a mouse model of aGVHD inhibited tissue injury in target organs, increased FoxP3+CD4+CD25+ Tregs on Day 7, decreased serum TNF-α on Days 7, 14, and 21 after murine allo-HSCT and prolonged survival (20). Reduced plasma amino acid levels including glutamine have been characterized in patients after allo-HSCT and associated with systemic inflammation (21). Indeed in a retrospective study patients who receive glutamine supplementation had less clinically documented infection and 100-day mortality (22). In a murine multi-organ system model of cGVHD with bronchiolitis obliterans, GLS knockout (KO) T cells were unable to cause disease (18). Th17 cells support germinal center reactions, a hallmark of disease in this cGVHD model and pharmacological inhibitors (RORγt small molecules or anti-IL17 neutralizing antibody) against an activated Th17-prone T cell subset mitigated disease. Anti-IL-17 antibodies have been FDA approved. A small molecule GLS inhibitor, CB-839, is in advanced clinical testing as an anti-cancer agent (23, 24) and could be repurposed to treat cGVHD. In contrast, local (oral) glutamine delivery has been shown to reduce treatment-related mucositis in patients with cancer, presumably through its antioxidant effects (25).

### Reactive Oxygen Species

NAD is a key coenzyme involved in metabolic pathways including glycolysis, TCA cycle, OXPHOS, FAO and one carbon metabolism (serine biosynthesis) that must be continuously replenished (26). Under inflammatory conditions and cellular stress, the NAD synthesizing enzyme nicotinamide phosphoribosyl transferase (NAMPT), the rate-limiting enzyme in the NAD salvage pathway, was upregulated enabling increases in intracellular NAD levels. NAMPT regulates the activity of various NAD dependent enzymes such as poly ADP-ribose polymerases, CD38, CD73, and sirtuins (26). The levels of NAMPT were found to be elevated in acute GI-GVHD patients (27). Specifically, NAMPT expression was most pronounced in colonic CD3+ T cells of both mice and aGVHD patients (27). A small molecule NAMPT inhibitor, Fk866, enhanced cell cycle arrest at the G1 phase and increases p53 acetylation. *In vivo* administration of Fk866 ameliorated murine aGVHD by selectively inducing apoptosis of T effector cells while sparing Tregs and memory T cells important for pathogen and tumor clearance. Further, Fk866 downregulated gene expression of IFN-*γ* and TNF-α in T conventional cells, inhibited Th17 differentiation and promoted Treg Foxp3 expression and lineage stability. Consistent with the murine model results, Fk866 inhibited the proliferation of human T cells from healthy and GVHD patients and promoted both *in vitro* induced Treg (iTreg) and *in vivo* Treg generation. Since Fk866 maintained GVT activity against leukemia, immunometabolism strategies that inhibit NAMPT would have advantage over more global immunosuppressants.

Sirtuin-1 (Sirt-1) is a member of the class III family of histone deacetylases (HDACs) and has been shown to modulate cellular metabolism by acting as a cellular sensor (28, 29). Sirt-1 is active in both the nucleus and the cytoplasm, and its targets are key regulators of various metabolic pathways. Sirt-1 inhibits T cell activation and the differentiation of Th1 and Th17 cells (30, 31). A recent study reported that Sirt-1 deficient T cells have impaired potential to produce IFN-γ and induce murine aGVHD (31). Deficiency of Sirt-1 promoted Treg differentiation and stability in aGVHD recipients. The effect of Sirt-1 inhibition was extended beyond the aGVHD models as the small molecule Sirt-1 inhibitor, Ex-527, prevented and reversed cGVHD. Mechanistic studies revealed that Sirt-1 deficiency reduced Tfh cell differentiation and reduced B cell activation and plasma cell differentiation. Attenuation of cGVHD with preservation of GVT was also observed in cGVHD mice treated *in vivo* with Ex-527 (31) that is in phase II clinical trials (32). Given its desirable properties discussed above, Ex-527 may be a candidate for clinical trials in GVHD.

The initiating events for acute and chronic GVHD start with the release of DAMPs from the conditioning regimen (1, 2). The DAMP ATP is released during necrosis which can activate the purinergic P2X7 receptor on APCs leading to subsequent activation of allo-reactive T cells (33). Compared to control mice, immune deficient NSG mice injected with human peripheral blood mononuclear cells to induce GVHD had increased murine P2RX7 in the duodenum, ileum, and skin (33). In addition to APC activation, P2X7R is required for the establishment, maintenance, and functionality of central and tissue resident memory T cells (34). Mechanistically P2X7R has been shown to promote mitochondrial homeostasis and metabolic function (34). During tissue damage, free DNA is also released, and high levels of free plasma mitochondrial DNA (mtDNA) have been associated with the onset of cGVHD (35). Plasma cell free mtDNA, measured from 39 adult patients post allo-HSCT with and without cGVHD (35), was found at significantly higher levels in cGVHD patients. These data correlated with B cell responsiveness to a TLR9 agonist, as shown by CD86 upregulation and known cGVHD biomarkers such as CXCL10, ICAM-1, CXCL9, sCD25 and sBAFF (35). Previous clinical trials have tested a TLR7,8, and 9 antagonist (IMO-8400) (36), offering the possibility that IMO-8400 or other TLR7,8, and 9 inhibitors could be repositioned for GVHD purposes.

Pre-conditioning regimens can exacerbate GVHD by increasing ROS and free radicals as well as reducing antioxidants. Upon allo-stimulation, donor T cells exhibited increased ROS production (37). Mitochondria derived ROS has been shown to be essential for T cell activation and proliferation (38, 39). Hence, multiple studies have attempted to prevent GVHD by reducing oxidative stress and maintaining redox balance in allo-HSCT preclinical models. Mitochondrial HDACs such as Sirt-3, the major mitochondrial sirtuin, control ROS production by promoting antioxidant scavenging mechanisms (40). Unexpectedly, loss of Sirt-3 in donor T cells attenuated aGVHD and retained the GVT response (41) rather than aggravating aGVHD. Mechanistically, Sirt-3 deficiency led to reduced ROS production in both non-specific TCR and allo-stimulated T cells, which may be indirectly due to impaired donor T cell activation. Interestingly, NAD+ and its cofactor nicotinamide compete with each other (42); nicotinamide has been shown to have clinical efficacy in cancer trials (43).

The redox master regulator nuclear factor (erythroid derived 2) factor 2 (Nrf2 or NFE2L2) is a transcription factor that maintains metabolic homeostasis by promoting antioxidant responses and anti-inflammatory responses. Experimental evidence showed that Nrf2 maintains immune tolerance and mitigates inflammation. While Nrf2 deficiency accelerated autoimmune diseases (44, 45), Nrf2 activation attenuated auto-inflammatory responses (46). However, studies of Nrf2 in murine allo-HSCT models have yielded conflicting results as Nrf2-deficient donor T cells induced less aGVHD morbidity and mortality. In contrast, sulforaphane an aliphatic isothiocyanate that activates Nrf2, ameliorated aGVHD (47, 48). In both models, GVT responses were preserved (48, 49) and the frequency of Tregs was increased. A recent study compared the expression pattern of Nrf2 on CD3+ T cells between allo-HSCT patients and healthy controls. Elevated expression of Nrf2 on both CD4+ and CD8+ T cells was observed in allo-HSCT patients, especially at time periods of cellular stress early post transplantation, then steadily declined over time and high Nrf2 expression in CD8+ T cells was associated with reduced cGVHD (50). Other studies also have explored the strategy of scavenging ROS to counteract oxidative stress by either administering NecroX-7, a necrosis inhibitor with an antioxidant mechanism, or by overexpressing antioxidant enzyme thioredoxin (51). Administration of NecroX-7 significantly improved aGVHD recipient survival which was correlated with the reduced levels of ROS and increased frequency of Tregs (51, 52). Donor allogeneic T cells overexpressing thioredoxin 1 had an impaired potential to induce aGVHD due to less ROS accumulation (52). This preclinical finding is promising as human recombinant Trx1treatment attenuated GVHD in both murine MHC mismatched and xenograft models, importantly without losing GVT responses (52).

### Lipid Metabolism

Activated alloreactive T cells in GVHD recipients have increased energy requirements to accommodate their expansile and effector functions (53). OXPHOS, shown to be utilized by T cells activated during GVHD, is the most efficient source of ATP. Multiple substrates can be used for OXPHOS. Upregulated FA transport, FAO enzymes, rates of FAO and transcriptional coactivators (53) during GVHD support the use of FA as the principal fuel source for alloreactive T cells (37). Pharmacological blockade of FAO by *in vivo* administration of etomoxir, a competitive inhibitor of CPT1a, resulted in decreased survival of donor alloreactive T cells without affecting T cells during normal immune reconstitution (53). Whereas BM cells from mice reconstituted by BM cells without T cells had increased aerobic glycolysis, alloreactive T cells in GVHD mice increased aerobic glycolysis and OXPHOS as well as accumulated acylcarnitines, indicating high FAO rates (37). These data suggest that inhibitors of OXPHOS or FAO may reduce GVHD without compromising hematopoietic reconstitution.

Lysosomal lipase (LAL), an intracellular lipase, hydrolyzes cholesteryl esters and triglycerides to produce free FAs and cholesterol. LAL is required for T cell development, maturation, activation, and function (54, 55). Notably, LAL deficiency in CD4+ T cells impaired pathogenic Th1 differentiation and increased Treg generation (54, 55). Loss of LAL compromised metabolic homeostasis and immune function (56). Yu and colleagues used both genetic and pharmacological approaches to inhibit LAL in an aGVHD model. Donor T cell LAL deficiency were defective in aGVHD induction as a result of lower survival, migration potential and metabolic function (57). LAL-deficient CD4+ donor T cells exhibited decreased CPT1a expression and higher oxidative stress levels, consequentially increased lipid content. Although pharmacological inhibition of LAL using orlistat diminished aGVHD, GVT responses were maintained (57).

The energy sensor AMPK promotes FAO and mitochondrial biogenesis (12). Metformin activates heterotrimeric AMPK (58). *In vivo* metformin administration increased the ratio of Treg/Th17, enhanced autophagy and reduced mTOR/STAT3 signaling (59) resulting in the amelioration of murine aGVHD severity (59). In contrast, a recent study reported that the loss of AMPK in donor T cells attenuated aGVHD (60), suggesting a pAMPK independent mechanism of GVHD amelioration by metformin (60). Allogeneic murine and human T cells upregulated pAMPK during early aGVHD and xenogeneic GVHD respectively. Donor T cell AMPKα1/α2 deletion (AMPK KO) attenuated aGVHD in each of two distinct murine models without compromising GVT responses. Reduced aGVHD was due to decreased AMPK-KO donor T cells. Surprisingly, no difference was observed in the canonical AMPK-related pathways of FAO, autophagy, or mTOR signaling between donor AMPK-KO and wildtype (WT) T cells. Future studies are warranted to consider metformin in preventing and treating GVHD.

## Modulating Intestinal Metabolism

### Intestinal Epithelial Cells

Alterations in the intestinal microbiota has been implicated in multiple diseases including GVHD (61). In aGVHD and cGVHD intestinal damage and microbial dysbiosis are central to pathogenesis (1, 2). Indeed, intestinal epithelial cell (IEC) damage has been shown to contribute to alloimmune and autoimmune diseases such as inflammatory bowel disease (IBD) and GVHD (62, 63). IECs form mucosal and chemical barriers including antimicrobial peptides to protect the host from invading pathogens (64). A recent study investigated the metabolic changes of IECs in aGVHD mice (62). Oxygen consumption rates (OCR), an indicator of OXPHOS, in allogeneic IECs (allo-IECs) were significantly lower from syngeneic IECs (syn-IECs) controls. Mitochondrial TCA cycle metabolite composition in IECs obtained from GVHD mice revealed high levels of succinate with low levels of succinate dehydrogenase A (SDHA), a component of mitochondria respiratory complex II. SDHA links the TCA cycle with the ETC by catalyzing the oxidation of succinate to fumarate in TCA and donating electrons to the ETC (65, 66). SDHA loss in allo-IECs was mediated by donor T cell cytotoxic molecules granzyme B and perforin. Specific deletion of SDHA in allo-IECs aggravated aGVHD related mortality. SDHA expression in intestinal biopsies was significantly lower in the colon of gastrointestinal (GI) GVHD patients than those without histologically GI GVHD. Modulating IEC metabolism to sustain or replenish or replace SDHA and favor OCR in the context of GI GVHD would represent a novel treatment strategy.

### Immunomodulatory Function of Microbial Metabolites

Allo-HSCT results in intestinal microbiota dysbiosis due to the conditioning regimen, immune attack and broad-spectrum antibiotic use (67). In the 1970s, the role of intestinal microflora in modulating GVHD was surmised from studies of germ-free mice exposed to aGVHD conditions (67). While germ-free conditions and antibiotics mitigated experimental GVHD (68, 69), clinical studies involving bacterial decontamination in allo-HSCT patients yielded mixed results (70–72). In more recent studies, aGVHD intestinal inflammation was associated with major shifts in intestinal microbiota with a loss of overall diversity, expansion of *Lactobacillales* and loss of *Clostridiales (* 73). *Lactobacillales* mediated significant aGVHD protection in mice and microbiota patterns in allo-HSCT patients mirrored those in mice. A retrospective study on 857 allo-HSCT patients reported that broad-spectrum antibiotics imipenem-cilastatin and piperacillin-tazobactam use increased GVHD mortality. A similar result was recapitulated in aGVHD mice treated with imipenem-cilastatin that had compromised intestinal barrier functions and loss of protective mucus lining (74). Microbes produce various metabolites from nutrients that influence intestinal immunity by acting as a bridge between microbes and the host immune system. Accumulating evidence suggests that microbial metabolites play a key role in tissue repair and immune regulation.

### Short Chain Fatty Acids

Short chain fatty acids (SCFA), primarily acetate, propionate, and butyrate, possess immunomodulatory properties that promote peripheral Treg generation (75), suppress Th17 generation (76) and modulate macrophage function (77). Butyrate, a nutrient source and an HDAC inhibitor, promotes IEC barrier function (78). Butyrate levels were significantly lower in the intestinal tissues of allo-HSCT mice due to the reduced IEC transporter expression and receptor activation. Increasing intestinal butyrate levels in allo-HSCT mice by oral administration of butyrate or bacteria that produce butyrate ameliorated aGVHD (79) that was associated with enhanced epithelial cell junctional integrity and function (79). Among SCFA sensors, the metabolic-sensor receptor, free-fatty acid receptor 2, was found to regulate IL-22-producing innate lymphoid cells (ILC3) that support intestinal stem cell proliferation and differentiation (80). The G-protein coupled receptor GPR43 that is activated by SCFAs proved critical for anti-GVHD effects mediated by butyrate and propionate and was reduced in allo-HSCT recipients (81). *Bacteroides fragilis* administration reduced murine acute and chronic GVHD lethality in allo-HSCT by improving gut integrity through increased levels of SCFA acetic and butyric acid (82). Consistent with mouse studies, SCFA were found to be reduced in 42 pediatric allo-HSCT patients who developed aGVHD (83). In another study involving 201 patients, a positive correlation between increased aGVHD mediated mortality and loss of butyrogenic bacteria was seen (84). Allo-HSCT patients who developed cGVHD had lower plasma concentrations of propionate and butyrate than controls (85). Although these findings support SCFA as a therapeutic to alleviate GVHD, a recent clinical study reported conflicting results as patients who had higher butyrogenic bacteria after gut GVHD were more likely to develop steroid refractory aGVHD or cGVHD (86). Butyrogens may have a protective effect against aGVHD onset but may aggravate the disease in patients with GI GVHD.

Chronic GVHD also is associated with GI dysbiosis with a loss of fecal microbiota diversity. In a case-control cohort of adult transplant patients, analysis of stool samples at various timepoints throughout allo-HSCT showed that the samples from cGVHD and control transplant patients were comparably diverse before allo-HSCT (day -30) and in the peri-engraftment period. At ~day +100, some patients continued to have dysbiotic microbial composition while others returned to a pre-transplant microbial composition with no significant differences between the cGVHD and control transplant patients (85). Shotgun metagenomic sequencing of day +100 stool samples yielded enrichment of the microbial metabolic pathways related to SCFA metabolism. Plasma concentrations of butyrate and propionate were significantly lower in cGVHD patients compared to control transplant patients (85). Since SCFAs are produced after microbial fermentation by anaerobic bacteria, a Bayesian logistic regression of stool samples revealed that the presence of the anaerobic genera *Lachnoclostridum*, *Clostridium* and *Faecalibactrium* were associated with a reduced incidence of cGVHD (85). These studies demonstrate that alterations in the gut microbiome and the production of microbial metabolites such as SCFA have implications for GVHD pathogenesis and severity. A clinical trial of potato-based resistant starch ingestion during conditioning through day +100 after allo-HSCT as a source of SCFA is in progress (ClinicalTrials.gov Identifier: NCT02763033).

### Long Chain Fatty Acids

Recent studies investigated the role of long chain fatty acids (LCFA) such as palmitic acid (PA) and stearic acid (SA) in modulating the pathogenesis of aGVHD (87, 88). Wu et al. conducted a study of serum collected from 114 allo-HSCT patients and found that the ratio of SA/PA metabolite could be an excellent biomarker in the allo-HSCT recipients to predict both aGVHD and relapse (87). Patients with lower SA/PA ratio were more likely to develop grade II–IV aGVHD than those with higher SA/PA ratios (87). To further examine the role of SA or PA in the development of GVHD, allo-HSCT mice were either fed with high PA or SA diet (88). A high PA diet neither protected nor aggravated aGVHD lethality, in contrast to a high SA diet that resulted in the enrichment of *Akkermansia* genera, specifically *A. muciniphila*, and aggravated aGVHD severity. Fecal metabolomes revealed increased SCFA acetate, butyrate, and propionate in recipients fed a high SA diet as compared controls. *A.muciniphila* or acetate administration aggravated aGVHD mortality in control fed recipients, suggesting that the gut microbiota shift and associated SCFA metabolites (mainly acetate) modulate aGVHD pathogenesis. In line with the murine findings, higher concentrations of *A. muciniphila* and acetate were found in aGVHD patients than those of non-GVHD controls (88). Further studies are required to understand the role of other LCFA and their associated metabolites in regulating GVHD lethality.

### Amino Acid and Vitamin Derived Metabolites

Indoles are either derived from plant food or microbial metabolites of dietary tryptophan. Similar to SCFA, indoles support intestinal barrier function by engaging with aryl hydrocarbon receptors and promoting IL-22+ ILC3 cell maintenance (89, 90). Colonization of the intestines of allo-HSCT mice with indole-producing bacteria reduced pathology, attenuated aGVHD and improved survival (91). Oral gavage of indole-3-carboxaldehyde (ICA), an indole derivative, ameliorated aGVHD while not abrogating donor T cell mediated GVT responses. Microbial metabolites can also promote pro-inflammatory milieu and aggravate aGVHD. Wu et al. reported that a choline rich diet or choline metabolite trimethylamine *N*-oxide (TMAO) accelerated murine aGVHD lethality by inducing M1 macrophage polarization *via* the inflammasome component NLRP3 (92). Reducing TMAO level by treating allo-HSCT mice with a trimethylamine (TMA) lyases inhibitor effectively controlled choline diet-induced aGVHD. Likewise, taurine, a metabolite of bile acid, has been shown to activate NLRP6 inflammasome signaling to promote pro-inflammatory cytokines in allo-HSCT recipient mice and thus exacerbate aGVHD (93).

Mucosal-associated invariant T (MAIT) are innate-like T cells that produce large amounts of cytokines such as IL-17A in response to bacteria and yeast through recognition of riboflavin metabolites presented by the MHC class I–like molecule MR1 (94). Hill and colleagues found that recipient MAIT cells reduced aGVHD by promoting intestinal barrier function, regulating microbial diversity, and suppressing donor alloantigen presentation and T cell expansion while driving Th1 and Th17 cells in the colon post-allo-HSCT (95). Chronic GVHD patients had a reduced number of MAIT cells compared to those without cGVHD possibly due to gut microbiota changes in cGVHD patients, including alterations of species required for the expansion of MAIT cells (96). A recent study reported that MAIT cells may be used as universal cells for cellular therapy due to their lack of alloreactivity and potency in causing xenogeneic GVHD (97).

Polyamines, cationic biogenic amines are derived from dietary arginine by both host and microbes (89). A study involving two cohorts of 43 and 56 patients reported that polyamine metabolites N-acetyl putrescine and N-acetyl spermidine were increased in allo-HSCT patients without GVHD (98). These metabolites have been shown to inhibit T cell (99) and pro-inflammatory macrophage activation (100) with an IEC protective role (101). Future investigation of the roles of microbial metabolites in clinical settings will provide more insight into their contributions to the pathogenesis of GVHD. Overall, altering the diet or microbiome to promote the production of beneficial metabolites and reduce the level of unwanted metabolites are a viable avenue to reduce GVHD.

## Metabolic Effects of Coinhibitory Pathway Blockade and Cellular Therapy in Allo-HSCT

The inhibitory receptor, PD-1, inhibits glycolysis and promotes lipolysis and FAO (102, 103). PD-1 pathway blockade post-allo-HSCT augmented aGVHD in mice (104, 105) and patients (106). Increased programmed death ligand 1(PD-L1) expression was seen on donor T cells in mice and patients with aGVHD (107). PD-L1 KO donor T cells had enhanced apoptosis, diminished gut homing antigens, inflammatory cytokine expression, and a blunted aGVHD capacity without GVT loss (107). In GVHD mice, PD-L1 KO donor T cells had decreased glycolysis, OXPHOS, FAO, and glutaminolysis, along with increased ROS, likely contributing to the observed lower aGVHD lethality capacity.

Preclinical and clinical allo-HSCT studies have demonstrated that adoptive cellular therapy is an attractive option to reduce GVHD *via* restoring immune tolerance (108). Major hurdles hampering the wide clinical applications of cellular therapies are the requirement for expansion of low frequency regulatory cells to large numbers with retention of functionality and avoidance of plasticity in the inflammatory milieu of GVHD. Thus, studies have attempted metabolic reprogramming of regulatory cells to harness their potency and functionality in allo-HSCT settings. Among regulatory cells in controlling GVHD, Tregs have been extensively studied due to their capacity to suppress allo-immune responses. Acute GVHD patients had a lower Treg frequency with impaired stability than allo-HSCT patients without GVHD or healthy controls (109). The loss of stability due to the inflammatory milieu in allo-HSCT settings is partially dependent on the transcription factor STAT3 (110). Phospho-STAT3 inhibited peripheral Treg generation in murine aGVHD (110). Inhibition of STAT3 phosphorylation (pSTAT3) in human Tregs enhanced the suppressive capacity and stability of iTregs (111). Notably, pSTAT3- inhibited iTregs significantly reduced xenogeneic GVHD compared to vehicle control, while sparing donor GVT responses. Inhibiting pSTAT3 in iTregs induced a shift toward glycolysis by inhibiting OXPHOS (111). Metabolic reprogramming of pSTAT3- inhibited iTregs with Coenzyme Q10 treatment enhanced their suppressive capacity by elevating basal and restoring the maximal spare capacity (111). Inhibiting protein kinase C-theta increased suppression of thymic-derived Tregs, reduced mTORC2 signaling, increased OCR and upon adoptive transfer *in vivo*, decreased aGVHD mediated GI damage (112).These results support the concept that metabolic reprogramming of Tregs can be of therapeutic value to treat GVHD.

Lipid metabolism coordinates Treg proliferation and survival (113, 114). Liver kinase B1 (LKB1), a serine/threonine kinase, regulates cell growth and lipid metabolism (115, 116). Loss of LKB1impaired Treg function and stability (116–118). Tregs from aGVHD patients expressed lower LKB1 gene and protein expression than controls (109). Extending the clinical findings to a murine allo-HSCT model, the adoptive transfer of LKB1 deficient Tregs failed to control aGVHD (109). LKB1 overexpression in human Tregs partially rescued Foxp3 expression that regulates Treg stability and function. Acetyl-CoA carboxylase 1 (ACC1) catalyzes the first step in *de novo* FAS (119). Selectively deleting ACC1 in Tregs or treating Tregs *ex vivo* with an ACC1 inhibitor enhanced *in vitro* suppressive capacity and increased oxidative and glycolytic metabolism (120). Adoptive transfer of ACC1 KO Tregs reversed established cGVHD in a multi-organ system model with bronchiolitis obliterans. These studies suggest that modulating Treg lipid metabolism by either overexpressing LKB1 or employing FAS inhibitor may be a useful strategy to treat GVHD.

Over the past two decades, attention placed on testing of the adoptive transfer of mesenchymal stem cells (MSCs) to treat GVHD (121, 122) has shown variable therapeutic efficacy (123–125) that was associated with their plasticity and metabolic fitness in response to the inflammatory milieu (126, 127). Priming cord blood derived MSCs with an *in vitro* inflammatory cytokine regimen reprogrammed MSC metabolism to exhibit increased glycolytic capacity and superior immunosuppressive capacity manifested as increased survival in allo-HSCT and xenogenic GVHD recipients (122).

Glycogen Synthase Kinase 3 (GSK3) is a serine/threonine (ser/thr) protein kinase and metabolic sensor that regulates glycogen metabolism, gene transcription, cell survival and signaling (128). One isoform, GSK3β, has been shown to promote murine and human iTreg generation (129). Treatment of B cells with GSK3β inhibitor enhanced regulatory B cells (Bregs) differentiation and suppressive function (130). Chronic GVHD patients showed a reduced frequency of Bregs than allo-HSCT patients without GVHD (130). In a xenogeneic GVHD model, adoptive transfer of *ex-vivo* purified Bregs treated with the GSK3β inhibitor improved survival and reduced target organ damage in GVHD mice (130). Collectively, these studies lay a foundation for future research in exploiting the metabolic pathways to potentiate regulatory cell function in controlling and treating GVHD.

## Conclusion

A growing number of preclinical allo-HSCT studies are pointing to the importance of immunometabolism in modulating alloreactive donor T cell responses to control GVHD and promote GVT. Metabolic intervention with pharmacological agents can harness regulatory cell potency and stability impairing pathogenic alloreactive donor T cell responses. Metabolic reprogramming of *ex-vivo* immune cells by gene editing technologies could be employed to target specific cell populations in an effort to enhance adoptive cellular therapy. A challenge in the field is the lack of clinical trials focused on metabolic interventions in GVHD. While there are retrospective metabolomic studies reporting correlative changes in host and microbiota-derived metabolites with aGVHD, prospective trials in various patient groups and treatment regimens are needed to identify metabolic pathways and targets for interventional trials. The clinicaltrials.gov website lists a limited number of clinical studies that have metabolism as one of the readouts. However, there is only one trial on clinicaltrials.gov (NCT02763033) specifically designed to prospectively alter metabolism for aGVHD prophylaxis and none are listed for aGVHD therapy or cGVHD prophylaxis or therapy. In the aGVHD prophylaxis study, allo-HSCT patients are being given potato-based resistant starch capable of increasing butyrate levels within the intestines to reduce rates of aGVHD (79). Future studies should focus on unraveling the relationship between metabolism and GVHD and of the metabolism-microbiota axis in order to select appropriate targets for intervention and to assess the safety and long-term effects of such metabolic interventions on infection risk and GVL in allo-HSCT clinical settings. Such deeper understanding of metabolic pathways and associated genes involved in immune cell dysregulation and non-hematopoietic cell damage during allo-HSCT should pave the way to provide novel therapies to prevent and treat GVHD.

## Author Contributions

FM and GT reviewed the literature, drafted, and edited the manuscript. SR, PS, RO’C, JR, and BB reviewed, edited the manuscript draft, and critically revised the final manuscript. All authors contributed to the article and approved the submitted version.

## Funding

This work was supported by grants from National Institutes of Health, National Institute of Allergy, and Infectious Diseases R37 AI34495 (BB), R01 AI153124 (PS), R01 AI153167 (JR), and T32 AI007313 (FM, SR); National Heart, Lung, and Blood Institute R01 HL56067 (BB), R01 HL11879 (BB), R01 HL115114 (BB) and R01 HL136664 (JR); National Institute for Diabetes and Digestive and Kidney Diseases R01 DK 105550 (JR); National Cancer Institute P01 CA065493 (BB) and R01 CA226983 (RO); Lupus Research Alliance Innovator Award (JR); Canadian Institutes of Health Research Fellowship (GT).

## Conflict of Interest

BB receives remuneration as an advisor to Magenta Therapeutics and BlueRock Therapeutics; Research funding from BlueRock Therapeutics, Rheos Medicines, Equilibre biopharmaceuticals, Carisma Therapeutics, Inc., and is a co-founder of Tmunity Therapeutics. PS receives remuneration for consulting to Immusoft, Merck, Velox Therapeutics and Takeda Pharmaceuticals, and receives grant support from Merck, Sanofi Genzyme, and Jounce Therapeutics. JR is a founder, scientific advisory board member, and stockholder of Sitryx Therapeutics, a scientific advisory board member and stockholder of Caribou Biosciences, a member of the scientific advisory board of Nirogy Therapeutics, has consulted for Merck, Pfizer, and Mitobridge within the past three years, and has received research support from Incyte Corp., Calithera Biosciences, and Tempest Therapeutics.

The remaining authors declare that the research was conducted in the absence of any commercial or financial relationships that could be construed as a potential conflict of interest.

## Publisher’s Note

All claims expressed in this article are solely those of the authors and do not necessarily represent those of their affiliated organizations, or those of the publisher, the editors and the reviewers. Any product that may be evaluated in this article, or claim that may be made by its manufacturer, is not guaranteed or endorsed by the publisher.
